# Development of insecticide resistance in malaria vector *Anopheles sinensis* populations from Shandong province in China

**DOI:** 10.1186/s12936-015-0592-8

**Published:** 2015-02-06

**Authors:** Yuhua Dai, Xiaodan Huang, Peng Cheng, Lijuan Liu, Haifang Wang, Huaiwei Wang, Jingxuan Kou

**Affiliations:** Department of Medical Entomology, Shandong Institute of Parasitic Diseases, 11 Taibai Middle Road, Jining, 272033 Shandong Province P.R. China; Henan Entry-exit Inspection and Quarantine Bureau of P.R.C, 269 Jinshui Road, Zhengzhou, 450003 Henan Province P.R. China

**Keywords:** Insecticide resistance, *Anopheles sinensis*, Malaria, Threat

## Abstract

**Background:**

*Anopheles sinensis* is a major vector of malaria and among the dominant species in Shandong province of China. Insecticide resistance is an important threat to vector-borne disease control. However, there are only few reports about insecticide resistance of *An. sinensis* populations from Shandong province.

**Methods:**

From 2003 to 2012, six districts in Shandong province were selected as the study areas. Insecticide susceptibility bioassay were tested on F1 progeny of *An. sinensis* to 4% DDT, 0.05% deltamethrin, 0.15% cyfluthrin, and 5% malathion, using the standard WHO resistance tube assay.

**Results:**

The resistance status of *An. sinensis* showed a significant decrease in the mortality rates in DDT, deltamethrin and cyfluthrin during the past ten years. Whereas obvious increase of mortality to malathion was observed throughout the assay, ranging from 47.37% to 86.62%.

## Background

The mosquito *Anopheles sinensis* is a major vector of human malaria in southeast Asia [1-3], and among the dominant species in Shandong province of China [4,5]. In the early 1960s and 1970s, Shandong province used to be an endemic region of malaria, where two large-scale outbreaks had occurred with an annual infection of six million and four million, respectively. After implementation of malaria control strategy, the disease successfully came under control. The Chinese government, consequently, formulated a plan of action for the elimination of malaria and decided to achieve the ambitious goal of eliminating malaria across China by 2020. However, recent malaria monitoring revealed a trend of increase in the infection of malaria in some areas of Shandong province mainly due to the cross-border migration.

In order to combat malaria, vector control is still indispensable in endemic foci [6]. Hence, vector surveillance is essential to prevent recrudescence of malaria in low-transmission regions [7]. Insecticides remain the most important vector control method. However, resistance to multiple classes of insecticides is becoming a common problem for malaria vectors and a serious threat to vector-borne disease control. There have been a number of reports about insecticide resistance of *An. sinensis* in China [8-14]. There have only been few articles about *Anopheles* resistance to insecticides in Shandong province. In this study, the extent and the development of insecticide resistance in *An. sinensis* were examined against four common insecticides recommended by WHO for malaria vector control by indoor residual spraying (IRS).

In China, organochlorines, such as DDT, have been intensively administered for malaria control during the 1950s. These compounds have progressively been replaced by alternative, more specific and less toxic chemicals, such as pyrethroids. Nowadays, pyrethroids have been extensively used inside houses and impregnated-bed nets for malaria control, as authorized by WHO [15]. Since 1997, at the national level, 2.5 × 108 kg insecticides have been used as active ingredients, annually. The area treated with pyrethroids occupied more than forty percent of the total insecticide-treated area in China [9]. However, the uncontrolled use of chemical insecticides not only leads to the environmental pollution, but also the emergence of resistance in *An. sinensis* populations. Insecticide resistance is a source of great concern and needs to be monitored in order to maintain the efficacy of vector control operations in the field. The purpose of this study was to determine the trends in insecticide resistance in *An. sinensis* from Shandong province, thereby providing guidance for insecticide use for malaria risk reduction.

## Methods

### Study area

**Figure 1:**
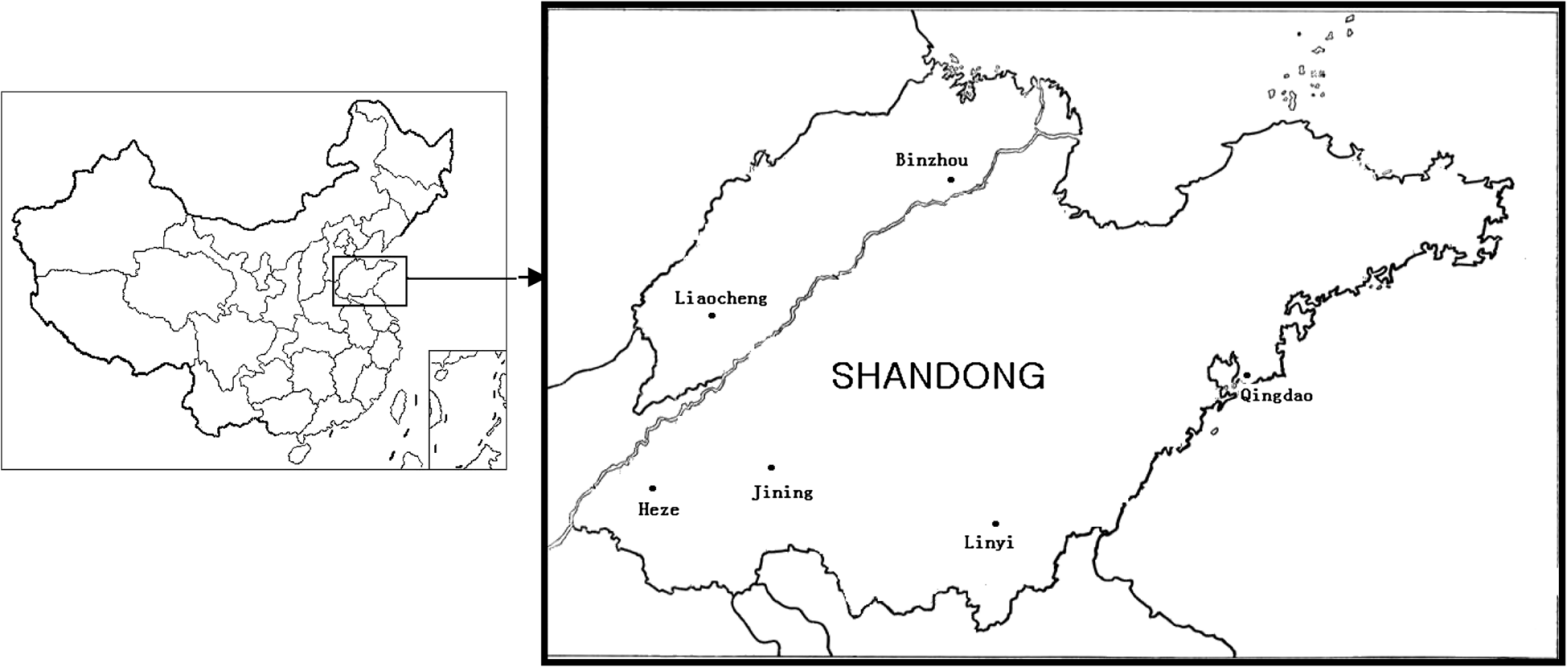
**The location of the mosquito collection from Shandong province in China.** The bars in the right map indicate 6 districts respectively.

The study was conducted in malaria endemic sites in Eastern China from 2003 to 2012. Six separate districts (Figure 1) of Shandong Province (114-112°E, 34-38°N) were involved. Each district survey is composed of cities, counties, and a number of villages. In the selected district, the sites were chosen to encompass a range of insecticide selection pressures including a highly urbanized area where inhabitants use insecticides against arthropod nuisance, a rural site with high coverage of pesticide application and a site with less insecticide usage.

### Mosquito sample collection

Each year, surveys were conducted during the summer and fall months when mosquitoes were most abundant and diverse. Adult mosquitoes were collected by different collection methods including indoor and outdoor human landing collection, collection on cattle and morning resting collections inside houses. *Anopheles sinensis* was not the dangered and protected species, so no specific permissions were required for these locations/activities. *Anopheles sinensis* adult mosquitoes were given fresh 8% sucrose solution daily. *Anopheles sinensis* mosquito larvae and pupae were collected from irrigated rice fields and small ponds with aquatic plants, using standard 350-ml dippers. All the mosquitoes collected were identified using morphological keys [16,17] and they all belonged to *An. sinensis* complex. Adults reared to the F1 families were tested to minimize the influence of mosquito age and blood feeding history in this study.

### Insecticide susceptibility bioassay

*Anopheles sinensis* female adult mosquitoes at 2–3 days post-emergence were used for susceptibility to four insecticides (4% DDT, 0.05% deltamethrin, 0.15% cyfluthrin, and 5% malathion), using the standard WHO resistance tube assay [18]. Bioassays were performed at a temperature ranging from 26°C to 28°C with 70% relative humidity. For each insecticide, a total of 100 approximately female mosquitoes were tested in insecticide susceptibility bioassays, with 20 mosquitoes per tube. A laboratory susceptible strain maintained in the insectary for more than 120 generation with no insecticide exposure was used as a susceptible mosquito control. Susceptible mosquitoes were exposed to the corresponding control papers impregnated with coal oil (DDT control), silicone oil (deltamethrin control), acetone oil (cyfluthrin control), and olive oil (malathion control). After insecticide treated for an hour, mosquitoes were transferred to recovery cups and maintained on 8% sucrose solution for 24 hrs, then the quantity of surviving mosquitoes was counted. If control mortality was between 5 and 20%, then the observed mortality was corrected according to the mortality rates of the respective control groups using Abbott’s formula following the WHO test procedures [19]. If the control mortality was below 5%, it was ignored and no correction was necessary. If the control mortality was above 20%, the tests were discarded. The bioassay results were summarized in three resistance classes as defined by WHO, susceptible if mortality was 98% or higher, possible resistant if mortality was 80–97%, and resistant if the mortality was less than 80% [20].

## Results

This is the first investigation of malaria vectors susceptibility to insecticides in Shandong province recently. The susceptibility of tested mosquitoes to 4% DDT, 0.05% deltamethrin, 0.15% cyfluthrin, and 5% malathion from 2003–2012 was presented in Table 1. The *An. sinensis* susceptible strain was susceptible to four insecticides. Abott’s formula was used to correct mortality rates of the sample with mortalities in the control group were 5-20%. No correction was required for the others populations, mortalities in control groups being below 5%.

**Table 1:** **Mortality rate and resistance status in a batch of** ***An. sinensis*** **in 10 replicates exposed to four insecticides**

**Year**	**DDT**			**Deltamethrin**			**Cyfluthrin**			**Malathion**		
	**N**	**Mortality%**	**Resistance status**	**N**	**Mortality%**	**Resistance status**	**N**	**Mortality%**	**Resistance status**	**N**	**Mortality%**	**Resistance status**
2003	101	58.93	R	96	81.37	M	102	80.60	M	108	47.37	R
2004	108	45.48	R	103	84.17	M	106	73.04	R	117	48.34	R
2005	99	33.37	R	104	73.58	R	112	63.29	R	105	56.52	R
2006	125	31.43	R	120	58.33	R	107	60.54	R	102	60.78	R
2007	117	38.54	R	115	58.50	R	114	47.55	R	107	69.16	R
2008	113	47.67	R	108	56.48	R	103	40.90	R	114	75.21	R
2009	107	47.31	R	104	50.22	R	118	33.05	R	120	77.11	R
2010	116	40.11	R	107	47.74	R	106	38.68	R	116	75.00	R
2011	115	30.43	R	113	44.25	R	100	30.77	R	103	81.60	M
2012	121	30.40	R	101	35.80	R	109	32.40	R	108	86.62	M
**Mean**		40.37			59.04			50.08			67.77	

N: Total number of mosquitoes tested.

Overall, the resistance status of *An. sinensis* showed a significant decrease in the mortality rates in DDT, deltamethrin and cyfluthrin during the past ten years. The corrected mortality rates were all below 80% for 4% DDT tested (Table 1). According to WHO criteria [20], *An. sinensis* mosquitoes from Shandong province were resistant to DDT for 10 years at least with a reduced mortality from 58.93% to 30.40%. From 2007 to 2009, DDT showed a transitory increase for mortality to mosquitoes, but expressed crosscurrent for the following four years.

The populations showed obvious reduction in susceptibility to both deltamethrin ranging from 81.37% to 35.80% and cyfluthrin from 80.60% to 32.40%. In the beginning of the study, the resistance level of the *An. sinensis* to deltamethrin and cyfluthrin were evaluated as “M” level. There was a significant decrease of mortality for the following test, the highest percentage of mosquitoes surviving the WHO diagnostic doses were seen in 2012 for deltamethrin and 2011 for cyfluthrin. Conversely, fold increase of mortality to malathion was observed throughout the assay, ranging from 47.37% to 86.62%. The mosquitoes were resistant to malathion in the early years and became medium resistant from the year 2011. The ten year mean difference in mortality rate for mosquitoes exposed to DDT was 40.37% (95% CI = 23.63-48.33); deltamethrin 59.04% (95% CI = 40.23-70.02); cyfluthrin 50.08% (95% CI = 32.76-60.24) and malathion 67.77% (95% CI = 46.03-77.19).

## Discussion

The study demonstrated that field populations of *An. sinensis* from Shandong province developed high resistance to three insecticides tested, including DDT, deltamethrin and cyfluthrin. The resistance was extremely prevalent and sustained, as more than 40% of mosquitoes survived the diagnostic dose for resistance, and in some cases up to 70% of the tested mosquitoes survived the bioassay. It could be inferred preliminarily that under long-term high insecticide selection pressure, *Anopheles* mosquitoes had evolved a strong resistance to various insecticides.

For *An. sinensis*, malathion was an effective insecticide in this study. From 2006, malathion was more effective than the others, as *Anopheles* mosquitoes treated by malathion exhibited a significantly higher mortality rate than the population for the other three insecticides tested.

This investigation displayed a significant reduction in the mean mortality rate for DDT, deltamethrin and cyfluthrin over the 10 year-period, indicating the existence of knockdown resistance in *An. sinensis* in Shandong province. Decrease in the mean mortality rates to pyrethroid (deltamethrin and cyfluthrin) and organochlorine (DDT) was interrelated, which agreed with some recent studies on *An. sinensis* in China [21-24]. Although the 24 h post-exposure mortality rate using DDT showed a fewer decrease compared to deltamethrin and cyfluthrin, the overall reduction in the mean mortality rate across all the three insecticides over the 10-year period suggested cross-resistance to pyrethroid and organochlorine.

It is known that pyrethroid insecticides are neurotoxins and share many characteristics with DDT including a negative temperature coefficient, a rapid knockdown effect followed by a lethal effect [25]. Furthermore, knockdown resistance (*kdr*) is a type of target-site resistance arising from point mutations in the sodium channel genes of the insect nervous system and is one of the mechanisms to confer cross-resistance to DDT and pyrethroids [26]. The resistance relationship between pyrethroid and DDT were also reported on *Anopheles gambiae s.s.* populations from Cameroon [27,28] and *Anopheles arabiensis* populations from Sudan [29,30]. From 1980s, DDT had been inhibited to be used on food supplies, fruits and vegetables in china. The mortality of *Anopheles* mosquitoes treated by DDT in 2012 was decreased obviously compared with the mortality in 2003. This test confirmed the correlation in mosquito resistance between pyrethroid and DDT.

Deltamethrin and cyfluthrin are the major pyrethroid insecticides for mosquito control and pest control in agriculture in Shandong province. The resistance to deltamethrin had risen significantly in the malaria-endemic areas compared to those in the 1990s [9]. The investigation revealed that the mortality to *An. sinensis* treated by deltamethrin decreased year after year for about nine years.

It has been suggested that insecticide resistance could be accentuated by the exposure of mosquito populations to pollutants and pesticides used in agriculture [31-34]. However, studies on *An. gambiae s.l.* have shown a strong correlation between resistance phenotype and *kdr* genotypes, announcing reduced susceptibility to pyethroid insecticides is mainly due to increased target site insensitivity caused by the *kdr* allele [35,36]. On the other hand, tests on *An. sinensis* determined that *kdr* mutations played a small role in resistance to some kind of insecticides tested including pyrethroid. Results provided in the study on *An. gambiae* revealed that *kdr* may act with certain co-factors that were thus far unidentified [30,37]. This resistance mechanism could be multigenic, and the *kdr* genotype might not fully explain all the variance in the resistance phenotype and the causal relationship between the *kdr* genotype and susceptibility to DDT and pyrethroids [38,39]. It has been reported that enhanced levels or modified activities of esterases and other detoxifying enzymes could prevent some insecticides from reaching their site of action [40]. The relationship between organophosphate resistance and high levels of esterases have been discussed in several studies [41,42]. Meanwhile, insensitive acetyl cholinesterase (AChE) is another common resistance mechanism to organophosphate insecticides [43]. Further understanding of the genetic basis of insecticide resistance is an essential step to implement more effective vector control strategies.

Since the mid-1980s, pyrethroids have been the dominant insecticides with pyrethroids-treated areas constituting more than one third of the total insecticide- treated area in China [9,44]. The rise in resistance of *An. sinensis* is probably facilitated by the intensive use of pyrethroids in agriculture, wood industry and public health in Shandong province. Malathion has been used to protect grain and control flies or cockroach but less extensively, resulting in the possible resistance in *An. sinensis.* In order to fight against mosquito resistance, pyrethroid insecticides with other types of insecticides in both public health and agricultural production need to be used restrictively and alternately.

This study shows for the first time the development of insecticide resistance in *An. sinensis* in Shandong province. However, there were some limitations in the study. Molecular confirmation of the existence of the *kdr* alleles and establishment the type of resistance were not done which means that the relationship between the development of the *kdr* mutations and the resistance could not established. At the same time, there is great need for continuous insecticide susceptibility testing on larval sampling in monitoring the efficacy of common insecticide and exploring the molecular basis of insecticide resistance since mosquitoes might develop different resistant mechanisms with different metabolic pathways in the larval and adult stages.
